# UV Protection Habits and Preferences in Patients With Distinct Cutaneous Immune‐Mediated Inflammatory Diseases

**DOI:** 10.1111/phpp.70084

**Published:** 2026-03-15

**Authors:** Zita Álvarez‐Bobillo, Iranzu Barandika‐Urrutia, Tamara Gracia‐Cazaña, Yolanda Gilaberte‐Calzada

**Affiliations:** ^1^ Department of Dermatology Miguel Servet University Hospital, IIS Aragón Zaragoza Spain; ^2^ Faculty of medicine University of Zaragoza Zaragoza Spain

**Keywords:** immune‐mediated skin diseases, photoprotection, sun protection factor, UV protection

## Abstract

**Background:**

Achieving adequate UV protection can be particularly challenging in patients with cutaneous immune‐mediated inflammatory dermatoses (IMIDs), owing to the clinical characteristics and quality‐of‐life impact of these diseases.

**Objectives:**

To compare the UV protection habits and preferences of patients with cutaneous IMIDs, specifically considering expert recommendations for these diseases.

**Methods:**

This pilot cross‐sectional, observational and analytical cohort study recruited 120 participants (78 female, 42 male; mean age, 40.8 ± 15.39 years): 20 cases for each IMID (vitiligo, atopic dermatitis, alopecia areata, psoriasis and hidradenitis suppurativa) and 20 healthy controls. Study variables included eye and hair color, BMI, phototype, clinical severity scales for each IMID, the Dermatology Life Quality Index (DLQI) score and a study‐specific questionnaire on photoprotection preferences.

**Results:**

Daily photoprotection was reported by 40% of participants, with highest adherence (55%) in vitiligo patients. Hat use was generally low (24.17%), except among alopecia areata patients (40%). Overall, 63.3% of participants reported no sunburn in the last year, and 95% used an SPF > 15. Cream was the most commonly used SPF format, followed by spray. Oral photoprotection was known by 33.3% of participants, with highest use reported by vitiligo patients (45%). Compliance with UV protection recommendations for their specific disease was highest among vitiligo patients (70%), but did not exceed 10% for any other IMID.

**Conclusions:**

Educating patients with cutaneous IMIDs about appropriate UV protection, tailored to their specific skin disorder, treatments and preferences, is crucial to improve compliance and disease control.

## 1 Introduction

UV protection plays a fundamental role in maintaining skin health and preventing damage caused by UV exposure. Its importance extends beyond skin cancer prevention to include mitigation of photo‐aging, pigmentation disorders, photodermatoses, DNA damage and even immunosuppression [1].

Immune‐mediated inflammatory diseases (IMIDs) are a group of diseases characterized by an exaggerated immune response, which can manifest as a range of inflammatory skin manifestations. The most prevalent cutaneous IMIDs in Spain [2] are psoriasis, atopic dermatitis, hidradenitis suppurativa (HS), vitiligo, and alopecia areata, reason why our study has focused on them.

UV protection relies on intrinsic and extrinsic mechanisms [1, 3, 4, 5, 6, 7]. Intrinsic defenses include physiological responses such as melanin production, antioxidant activity, and DNA repair; however, these mechanisms are often insufficient: that is the reason why extrinsic UV protection is recommended. The latter comprises behavioral, physical, and chemical measures: avoidance of sun exposure during peak ultraviolet radiation hours, use of protective clothing, such as hats and sunglasses, broad‐spectrum sunscreens… [1, 3, 4, 5, 6].

Different research [1, 3, 8, 9, 10, 11, 12, 13] supports all these measures as effective strategies for both healthy populations and patients with immune‐mediated dermatoses. They are essential for individuals with psoriasis [8], vitiligo [9], atopic dermatitis [10, 11], hidradenitis suppurativa [12] and alopecia areata [13], particularly when undergoing phototherapy or if they are susceptible to UV‐induced exacerbations and adverse effects, although tolerability and adherence may vary depending on skin condition.

In addition to these measures, oral photoprotection has emerged as a complementary strategy [5, 14, 15, 16, 17]. Most oral photoprotective agents exert antioxidant effects, reducing oxidative stress and preventing molecular and DNA damage caused by ultraviolet radiation, infrared radiation, and visible light [14, 15]. A key advantage of oral photoprotection is its ability to protect the entire skin surface independently of application technique, water exposure, or reapplication, without interfering with cutaneous vitamin D synthesis [14, 15, 16, 17].

The most common ingredient in oral sunscreens is Polypodium leucotomos (PLE) extract, a naturally occurring extract rich in polyphenols, which gives it antioxidant activity. Polypodium leucotomos has been used in combination with phototherapy in patients with vitiligo to reduce photosensitivity during treatment, thus allowing treatment to continue and improving repigmentation [18, 19]. A review by Choudhry SZ et al. [18] summarized the role of oral administration of Polypodium leucotomos in various skin conditions, including vitiligo, psoriasis and atopic dermatitis. They highlight that the antioxidant effects of PLE play a relevant role in the pathophysiology of vitiligo, as its use as an adjunct to phototherapy could improve the response to treatment in terms of the speed and extent of repigmentation [18]. Regarding the use of PLE in psoriasis, it appears that adding this substance to PUVA treatment could minimize the adverse effects of this treatment, mainly those related to local immunosuppression and photocarcinogenesis. With regard to atopic dermatitis, a clinical trial conducted by A. Ramírez‐Bosca [20] concluded that prolonged administration of oral PLE in patients with AD (added to first‐line treatment) could have benefits in terms of reducing the use of topical corticosteroids or oral antihistamines.

Overall, UV protection strategies show a favorable safety profile when appropriately used. Physical measures are safe and cost‐effective [21], while topical and oral photoprotective agents are generally well tolerated. Nevertheless, high‐quality data on long‐term outcomes and comparative cost‐effectiveness, particularly in specific IMIDs, remain limited and warrant further investigation.

Emphasizing personalized and targeted approaches is essential to promote adequate UV protection. Tailored UV protection is particularly important for individuals with these conditions: while UV exposure can exacerbate some cutaneous IMIDs, others may benefit from carefully managed UV therapy. In psoriasis, sun exposure reduces keratinocyte proliferation and T‐cell infiltration, decreasing inflammation and improving skin lesions [8, 22, 23]. With regard to atopic dermatitis [10, 11], narrowband UVB phototherapy is recommended as a therapeutic option, as it reduces itching and severity and improves sleep with a favorable safety profile [24, 25]. On the other hand, sun exposure and narrowband UVB phototherapy in vitiligo stimulate repigmentation by inducing melanocyte proliferation and migration [9]. It is the first‐line treatment and is associated with better repigmentation rates when combined with other treatments [26, 27, 28]. In addition recently, it has been reported that in alopecia areata UVB phototherapy can induce follicular repopulation and promote hair regeneration, especially if started in the early stages of the disease [29, 30, 31, 32]. However, hidradenitis suppurativa is the only condition in which there is no robust evidence of direct benefit from sun exposure or phototherapy on the disease, although synthesized vitamin D could have modulating effects on skin inflammation [33, 34, 35].

Therefore, individualized strategies are often required to address specific skin vulnerabilities and optimize patient outcomes [1].

A panel of experts, led by Passeron et al. [1], has issued specific UV protection recommendations for patients with each of these conditions. These recommendations underscore the need for individualized UV protection measures within this patient population, highlighting the link between effective prevention and improved quality of life [1].

Despite publication of these guidelines in 2021, no studies have comprehensively assessed the UV protection knowledge, habits, and preferences of cutaneous IMID patients. UV protection practices may vary greatly depending on the specific type of immune‐mediated inflammatory dermatosis affecting the individual, as well as the availability of sunscreen, which varies depending on each country. In Spain, these products are classified as cosmetics, so as they are excluded from national health coverage and must be paid for by the patient, which may hinder long‐term therapeutic adherence [21]. In addition, preferences may not necessarily align with expert recommendations [1, 21].

The primary objective of this study was to characterize UV protection habits and preferences in patients with different cutaneous IMIDs and to assess how these preferences align with expert recommendations.

## 2 Methods

This observational analytical cohort study included two cohorts: cutaneous IMID patients and healthy controls. The IMID population included patients diagnosed with psoriasis, atopic dermatitis, vitiligo, alopecia areata, or HS (*n* = 20 per condition). These patients were followed at the Dermatology Department of the Miguel Servet University Hospital (HUMS) in Zaragoza, Spain, between March and June 2024.

The control group consisted of healthy individuals recruited from companions of patients attending the Dermatology Department. Groups were approximately matched for age and sex. All participants provided informed consent prior to inclusion.

Sociodemographic variables collected included age, sex, eye color, hair color, body mass index (BMI) and phototype. Clinical severity scores for the different diseases (PASI, EASI, SALT, BSA, Hurley) were also recorded. Additionally, quality of life was assessed using the Dermatology Life Quality Index (DLQI) questionnaire [36].

All participants completed a validated photoprotection habits questionnaire, previously used by our research group [37], along with a photoprotection preference questionnaire and a questionnaire exploring adherence to disease‐specific UV protection recommendations. Both new questionnaires were specifically developed for this study (Supporting Information).

To ensure efficient data collection, questionnaires were administered to patients during their visits to the HUMS Dermatology Department throughout the study period.

### 2.1 Statistical Analysis

Quantitative variables were expressed as mean values and standard deviation (SD), and qualitative variables as absolute and relative frequencies. After assessing the distribution of the data, an independent‐sample Student's *t*‐test was used to compare mean values between study groups. Chi‐squared and Fisher's exact tests were used to compare proportions. Analysis of variance (ANOVA) was used to compare means across the six groups. A *p*‐value < 0.05 was considered statistically significant. Data analyses were performed using SPSS software (version 24.0; IBM Corp, Armonk, NY).

### 2.2 Ethical Committee Approval

The study was approved by the research ethics committee of the Autonomous Community of Aragon (CEICA), project p124‐130; report n° 07/2024.

## 3 Results

The study included 100 patients diagnosed with psoriasis (*N* = 20), atopic dermatitis (*N* = 20), vitiligo (*N* = 20), HS (*N* = 20) and alopecia areata (*N* = 20), along with a control group (*N* = 20), totalling 120 participants. The mean age of participants was 40.8 ± 15.39 years, and 65% were women.

Table 1 presents the demographic characteristics of the study population, including age, sex, phototype, hair color and eye color. There were no significant differences among the groups in age, sex, phototypes, hair color, or eye color. BMI was highest among patients with psoriasis and HS (*p* = 0.04).

**TABLE 1:** Study participants: Demographics, clinical severity, and impact of disease on quality‐of‐life.

	AA	HS	AD	Psoriasis	Vitiligo	Controls	Total	*p* Significant (*p* < 0.05)
Age (mean; SD)	(36.9; 12.63)	(35.25; 10.21)	(33.3; 17.69)	(42.55; 13.61)	(47.2; 17.06)	(47.1; 15.34)	(40.38; 15.39)	0.06
Sex (*n*, %)								
Women	(13.65%)	(13.65%)	(12.60%)	(13.65%)	(14.70%)	(12.60%)	(78.65%)	0.994
Men	(7.35%)	(7.35%)	(8.30%)	(7.35%)	(6.30%)	(8.30%)	(42.35%)	
Phototype (*n*, %)								
High risk I–II and III	18 (90%)	20 (100%)	19 (95%)	20 (100%)	12 (60%)	19 (95%)	108 (90%)	0.110
Low risk IV–V and VI	2 (10%)	0 (0%)	1 (5%)	0 (0%)	8 (40%)	1 (5%)	12 (10%)	
BMI (mean, SD)	(24.08; 4.46)	(28.71; 5.54)	(23.71; 4.01)	(27.43; 6.1)	(23.3; 3.35)	(25.26; 6.16)	(25.43; 5.37)	0.04
Hair color (*n*, %)								
Brown	(15.75%)	(15.75%)	(14.70%)	(15.75%)	(15.75%)	(14.70%)	(88.73%)	0.689
Blonde	(1.5%)	(2.10%)	(3.15%)	(3.15%)	(1.5%)	(5.25%)	(15.13%)	
Black	(4.20%)	(3.10%)	(3.15%)	(2.10%)	(4.20%)	(1.5%)	(17.14%)	
Eye color (*n*, %)								
Brown	(14.70%)	(17.85%)	(18.90%)	(14.70%)	(15.75%)	(10.60%)	(89.74%)	0.104
Green	(3.15%)	(1.5%)	(5.25%)	(2.10%)	(5.25%)	(6.30%)	(18.5%)	
Blue	(1.5%)	(2.10%)	(1.5%)	(4.20%)		(3.10%)	(10;8.3%)	
Black	(2.10%)						(3;2.5%)	
Severity (mean, SD)	SALT (32.25; 26.97)	HURLEY (2; 0.86)	EASI (9.12; 16.09)	PASI (8.56; 9.24)	BSA (30; 22.71)			
Severity								
Mild	10 (50%)	7 (35%)	14 (70%)	10 (50%)	3 (15%)		44 (44%)	0.616
Moderate	2 (10%)	6 (30%)	4 (20%)	4 (20%)	11 (55%)		27 (27%)	
Severe	8 (40%)	7 (35%)	2 (10%)	6 (30%)	6 (30%)		29 (29%)	
DLQI (mean, SD)	(4.60; 8.20)	(11.20; 5.50)	(7.00; 7.62)	(6.30; 6.70)	(7.55; 6.76)		(7.33; 7.21)	0.001

Abbreviations: AA, alopecia areata; AD, atopic dermatitis; BMI, body mass index; DLQI, Dermatology Life Quality Index; HS, hidradenitis suppurativa.

Disease severity was mild in most patients (44%, *n* = 44) (Table 1). Quality of life (mean DLQI score) was highest among HS patients (11.20 ± 5.50) followed by vitiligo patients (7.55 ± 6.76). Patients with alopecia areata had the lowest DLQI score (4.6 ± 8.2) (*p* = 0.001).

### 3.1 UV Protection Habits and Preferences

Table 2 details the UV protection habits and preferences of the study population. Daily sunscreen use was reported by 40% of all participants (*n* = 48). Adherence to this practice was highest among vitiligo patients (55%, *n* = 11), exceeding that observed in the control group (50%, *n* = 10), and was lowest among HS patients (20%, *n* = 4) (*p* = 0.242). Although sunscreen use varied, 95% of participants (*n* = 114) used products with a sun protection factor (SPF) > 15. Frequency of application varied significantly. Most participants applied sunscreen only once per day. However, 45% (*n* = 11) of vitiligo patients applied sunscreen daily (versus 40% of controls), while 55% (*n* = 11) of HS patients did not apply it on any day (*p* = 0.01).

**TABLE 2:** Participants' responses to the questionnaire on photoprotection habits and preferences.

	Psoriasis	Vitiligo	HS	AD	AA	Controls	*p* value Significant (*p* < 0.05)
Daily photoprotection							0.242
Yes (*n*, %)	(6.30%)	(11.55%)	(4.20%)	(9.45%)	(8.40%)	(10.50%)	
No (*n*, %)	(14.70%)	(9.45%)	(16.80%)	(11.55%)	(12.60%)	(10.50%)	
Number of times you have applied sunscreen in the last month							0.01
Every day (*n*, %)	(5.25%)	(9.45%)	(4.20%)	(3.15%)	(5.25%)	(8.40%)	
More than 20 days (*n*, %)	(0.0%)	(2.10%)	(0.0%)	(4.20%)	(0.0%)	(0.0%)	
More than 10 days but less than 20 (*n*, %)	(2.10%)	(4.20%)	(5.25%)	(0.0%)	(3.15%)	(2.10%)	
From 5 to 10 days (*n*, %)	(3.15%)	(3.15%)	(0.0%)	(2.10%)	(4.20%)	(3.15%)	
Less than 5 days (*n*, %)	(5.25%)	(1.5%)	(0.0%)	(4.20%)	(2.10%)	(6.30%)	
None (*n*, %)	(5.25%)	(1.5%)	(11.55%)	(7.35%)	(6.30%)	(1.5%)	
Number of times sunscreen is applied per day							0.032
Every 2 h (*n*, %)	(3.15%)	(3.15%)	(3.15%)	(2.10%)	(6.30%)	(4.20%)	
Twice a day (*n*, %)	(1.5%)	(8.40%)	(2.10%)	(3.15%)	(0.0%)	(5.25%)	
Once a day (*n*, %)	(16.80%)	(9.45%)	(15.75%)	(15.75%)	(14.70%)	(11.55%)	
Sun protection factor used							0.09
50+	(8.40%)	(9.45%)	(9.45%)	(12.60%)	(10.50%)	(9.45%)	
30–50	(9.45%)	(11.55%)	(10.50%)	(6.30%)	(10.50%)	(6.30%)	
16–29	(1.5%)	(0.0%)	(1.5%)	(2.10%)	(0.0%)	(2.10%)	
2–15	(2.10%)	(0.0%)	(0.0%)	(0.0%)	(0.0%)	(3.15%)	
Sunburns in the last year							0.127
None (*n*, %)	(15.75%)	(13.65%)	(15.75%)	(13.65%)	(10.50%)	(9.45%)	
1–2 (*n*, %)	(2.10%)	(4.20%)	(4.20%)	(5.25%)	(8.40%)	(6.30%)	
3–5 (*n*, %)	(3.15%)	(1.5%)	(1.5%)	(1.5%)	(2.10%)	(3.15%)	
6–10 (*n*, %)	(0.0%)	(1.5%)	(0.0%)	(1.5%)	(0.0%)	(2.10%)	
More than 10 (*n*, %)	(0.0%)	(1.5%)	(0.0%)	(0.0%)	(0.0%)	(0.0%)	
Sun exposure is considered beneficial for skin condition							0.001
Yes (*n*, %)	(19.95%)	(15.75%)	(11.55%)	(10.50%)	(6.30%)		
No (*n*, %)	(1.5%)	(5.25%)	(9.45%)	(10.50%)	(14.70%)		
Improvement with sun exposure							0.001
No, I even get worse (*n*, %)	(1.5%)	(2.10%)	(4.20%)	(4.20%)	(4.20%)		
No change (*n*, %)	(0.0%)	(7.35%)	(7.35%)	(8.40%)	(14.70%)		
Yes, I improve (*n*, %)	(19.95%)	(11.55%)	(9.45%)	(8.40%)	(2.10%)		
Habitual use of cap/hat							0.003
Yes (*n*, %)	(6.30%)	(6.30%)	(0.0%)	(2.10%)	(8.40%)	(7.35%)	
No (*n*, %)	(14.70%)	(14.70%)	(20.100%)	(18.90%)	(12.60%)	(13.65%)	
Sunscreen format used							0.140
Lotion (*n*, %)	(1.5%)	(3.15%)	(0.0%)	(1.5%)	(0.0%)	(0.0%)	
Spray (*n*, %)	(3.15%)	(1.5%)	(8.40%)	(2.10%)	(6.30%)	(3.15%)	
Cream (*n*, %)	(14.70%)	(14.70%)	(11.55%)	(14.70%)	(13.65%)	(12.60%)	
Gel (*n*, %)	(0.0%)	(2.10%)	(1.5%)	(0.0%)	(0.0%)	(0.0%)	
Oil (*n*, %)	(2.10%)	(0.0%)	(0.0%)	(1.5%)	(0.0%)	(4.20%)	
Stick (*n*, %)	(0.0%)	(0.0%)	(0.0%)	(2.10%)	(1.5%)	(1.5%)	
Preferred photoprotector format depending on your condition							0.004
Lotion (*n*, %)	(3.15%)	(1.5%)	(0.0%)	(2.10%)	(0.0%)		
Spray (*n*, %)	(8.40%)	(3.15%)	(10.50%)	(5.25%)	(13.65%)		
Cream (*n*, %)	(8.40%)	(10.50%)	(9.45%)	(11.55%)	(7.35%)		
Gel (*n*, %)	(0.0%)	(4.20%)	(1.5%)	(0.0%)	(0.0%)		
Oil (*n*, %)	(1.5%)	(2.10%)	(0.0%)	(1.5%)	(0.0%)		
Stick (*n*, %)	(0.0%)	(0.0%)	(0.0%)	(1.5%)	(0.0%)		
Discomfort when applying sunscreen							0.001
Yes (*n*, %)	(1.5%)	(3.15%)	(3.15%)	(8.40%)	(0.0%)	(0.0%)	
No (*n*, %)	(19.95%)	(17.85%)	(17.85%)	(12.60%)	(20.100%)	(20.100%)	
Knowledge of oral photoprotection							0.001
Yes (*n*, %)	(3.15%)	(15.75%)	(3.15%)	(3.15%)	(5.25%)	(11.55%)	
No (*n*, %)	(17.85%)	(5.25%)	(17.85%)	(17.85%)	(15.75%)	(9.45%)	
Use of oral photoprotection							0.001
Yes (*n*, %)	(0.0%)	(9.45%)	(2.10%)	(0.0%)	(0.0%)	(4.20%)	
No (*n*, %)	(20.100%)	(11.55%)	(18.90%)	(20.100%)	(20.100%)	(16.80%)	
Would like to use oral photoprotection							0.012
Yes (*n*, %)	(15.75%)	(20.100%)	(18.90%)	(17.85%)	(11.55%)	(11.55%)	
No (*n*, %)	(5.25%)	(0.0%)	(2.10%)	(3.15%)	(9.45%)	(9.45%)	

Abbreviations: AA, alopecia areata; AD, atopic dermatitis; HS, hidradenitis suppurativa.

Physical photoprotection measures, such as hat use, were infrequently practiced, with only 24.17% (*n* = 29) of participants reporting regular use. Adherence to this measure was highest among alopecia areata patients (40%, *n* = 8) (*p* = 0.03).

Analysis of sunscreen format preferences revealed that creams were the most commonly used and preferred format, favored by 65% of participants (*n* = 78). Sprays were the second most preferred format, particularly among HS (50%, *n* = 10) and alopecia areata (65%, *n* = 13) patients. Notably, 40% (*n* = 8) of atopic dermatitis patients reported discomfort with sunscreen application (*p* = 0.001).

Information on whether the sunscreen used contained mineral or chemical filters was not collected in this study.

We observed no significant differences in the number of sunburns reported in the preceding year across groups. However, the percentage of participants reporting no sunburns was lowest in the control (45%, *n* = 9) and alopecia areata (50%, *n* = 10) groups, and the highest in the psoriasis (75%, *n* = 15) and HS groups (75%, *n* = 15).

### 3.2 Awareness and Use of Oral Photoprotection

Knowledge about oral photoprotection was limited across all groups, with 66.7% of participants reporting no awareness of the concept. Use of oral photoprotection was highest among vitiligo patients (45%, *n* = 9).

### 3.3 Sun Exposure Perception in Cutaneous IMID Patients

Sun exposure was perceived as beneficial for their condition by all IMID patient groups, except for the alopecia areata group (Table 2, *p* = 0.001). 95% of psoriasis patients (*n* = 19) perceived sun exposure as beneficial and linked exposure to noticeable improvements in their condition. This was followed by vitiligo (55%, *n* = 11), HS (45%, *n* = 9) and AD (40%, *n* = 8) patients. Only 10% of alopecia areata patients (*n* = 2) reported a perceived benefit.

Table 3 illustrates the degree of compliance with expert‐agreed UV protection recommendations for patients with cutaneous IMIDs. The recommendations established for their condition were followed by 70% of vitiligo patients (*n* = 14) and 50% of alopecia areata patients (*n* = 10). All HS patients were aware of the need for photoprotection when using tetracyclines. Atopic dermatitis patients reported moderate adherence to their recommendations: 80% (*n* = 16) were aware that high temperatures and sweating can exacerbate injuries, and hence the recommendation to avoid such situations. No cases of allergic contact dermatitis to sunscreens were reported. Although 80% of psoriasis patients (*n* = 16) were aware that sunburn could worsen their disease, only 50% (*n* = 10) were aware that regular sun exposure is recommended for this group.

**TABLE 3:** Degree of compliance with expert‐agreed recommendations for photoprotection in patients with cutaneous IMIDs.

Cutaneous IMID	Questions	Answers (*n*, %)
Alopecia areata	Physical protective measures (e.g., hat, cap) and sun protection SPF 50+ are recommended for hairless lesions exposed to direct sunlight.	Yes: 10 (50%)
		No: 10 (50%)
Hidradenitis suppurativa	It is recommended to use SPF 50+ photoprotection to avoid post‐inflammatory hyperpigmentation in areas with lesions.	Yes: 8 (40%)
		No: 12 (60%)
	If individuals being treated with tetracyclines, adequate and rigorous photoprotection with broad‐spectrum sunscreen (SPF 50+) is recommended on a daily basis.	Yes: 6 (100%)
		No: 0 (0%)
Atopic dermatitis	To avoid systemic absorption and photosensitisation reactions, do not apply sunscreen until the lesions have been treated and the inflammation has resolved.	Yes: 6 (30%)
		No: 14 (70%)
	High temperatures and sweating can exacerbate lesions: it is recommended to avoid these situations.	Yes: 16 (80%)
		No: 4 (20%)
	Do not apply sunscreen to moist or oozing lesions, or lesions eroded by severe scratching.	Yes: 11 (55%)
		No: 9 (45%)
	Regular use of broad‐spectrum sunscreens (SPF 50+) is recommended.	Yes: 12 (60%)
		No: 8 (40%)
Psoriasis	Sun exposure can be beneficial in psoriatic patients: regular sun exposure is recommended.	Yes: 10 (50%)
		No: 10 (50%)
	Sun exposure should be limited and patients should avoid sunburn by applying sunscreen.	Yes: 16 (80%)
		No: 4 (20%)
Vitiligo	Patients with vitiligo are advised to regularly expose skin lesions to the sun without sunscreen, until the lesions start to turn pink. Subsequently, sunscreen (SPF 50+) should be applied to prevent sunburn.	Yes: 14 (70%)
		No: 6 (30%)

Abbreviation: SPF, sun protection factor.

## 4 Discussion

There is no official international consensus on recommendations for UV protection advice in patients diagnosed with dermatological IMID. This study highlights significant differences in UV protection practices among patients with these diseases. Our findings indicate that consistent use of daily sunscreen or oral photoprotection was uncommon among most patients and was most frequent among vitiligo patients. Overall, adherence to expert recommendations was generally low, with the highest and lowest levels of compliance observed for vitiligo and atopic dermatitis patients, respectively.

### 4.1 Comparison Between IMID Patients and Controls

Patients with cutaneous IMIDs have distinct UV protection habits and needs compared to healthy controls. While only 40% (*n* = 48) of all participants applied daily photoprotection, awareness and protective habits were greater among IMID patients, particularly those with more visible signs or conditions exacerbated by light exposure. The control group reported a higher incidence of sunburns in the preceding year, reflecting a lower perceived risk or concern associated with sun exposure.

Creams were the most commonly used form of photoprotection by both IMID patients and controls. However, there was a notable preference for spray formulations among patients with alopecia areata (65%, *n* = 13), HS (50%, *n* = 10) and psoriasis (40%, *n* = 8). This preference can be attributed to the ease of application and reduced skin contact offered by sprays, which is particularly important for patients with inflammatory lesions or those located in difficult‐to‐reach areas [2]. We observed a significant difference in photoprotector format preferences between groups (*p* = 0.004), suggesting that these choices are not random and may be linked to the clinical characteristics and practical needs of each group. Thus, while cream was the most widely used and preferred format overall, IMID patients show a greater inclination toward alternative formulations such as sprays, which may better suit their specific condition.

The preference for cream‐based sunscreens observed in our study aligns with established principles of barrier repair and allergen avoidance [1, 3, 38, 39]. A study developed by Xu et al. [38] analysed 174 top‐selling whole‐body moisturizers from major US online retailers, and found that lotions were the most popular vehicle, followed by creams, oils and butters. This study also underscores the importance in selecting suitable topicals, emphasizing that 12% of top‐selling moisturizers are free from common allergens such as fragrances, parabens and tocopherol. This discrepancy reveals a significant gap between commercial availability and the dermatological requirements of patients with altered skin barriers. Given that clinical guidelines [1, 39] prioritize bland, fragrance‐free formulations to minimize irritancy and optimize barrier restoration.

These differences should be carefully considered when designing personalized UV protection recommendations to improve adherence.

Our findings also indicated that awareness and use of oral photoprotection was greater among IMID patients, in particular those with vitiligo, compared to controls.

### 4.2 Recommendations Least Followed by Patients With Cutaneous IMIDs

This study underscores a persistently low level of compliance with several specific UV protection recommendations provided for patients with cutaneous IMIDs.

Daily use of sunscreen was the recommendation least followed by IMID patients, with only 40% of participants applying it regularly, despite undergoing treatment or having photosensitive skin conditions. This percentage of non‐daily sunscreen use (60%) is higher than that reported by Arteaga‐Henriquez et al. [40] in their study of photoprotection habits among kidney transplant patients (49.8%). Conversely, 95% of participants in our study (*n* = 114) used sunscreen with an SPF > 15, a higher percentage than that reported by García‐Malinis et al. [41] among mountain trail‐running athletes (61.9%).

Only 24.17% of the sample used hats or caps as a form of photoprotection, a significantly lower percentage than that obtained by García‐Malinis et al. [41], with 52.2%. Durán‐Ávila JJ et al. [42] conducted a study in which they examined the knowledge and habits regarding UV protection among Spanish and Italian medical students, in which 25.6% of participants always or almost always used a hat or cap as physical protection, while 46.1% never or almost never used one. Navarro Bielsa A. et al. [43] conducted a study comparing sun exposure habits and the use of photoprotection measures in patients diagnosed with different types of skin cancer. They found that melanoma patients were less likely to use clothing and shade to avoid sun exposure (*p* < 0.05), while BCC and SCC patients reported greater use of head coverings (*p* = 0.01).

Reapplication of sunscreen and appropriate frequency of use were other measures for which poor compliance was observed. Most patients, even those who used sunscreen, applied it only once daily, without reapplying during sun exposure, which compromises its effectiveness.

Awareness of oral photoprotection is also limited to only 33.3% of participants (*n* = 40). Despite the proven usefulness of some oral photoprotectors (e.g., *Polypodium leucotomos*) in conditions like vitiligo and atopic dermatitis [16, 44], the low level of usage among participants may be due to limited dissemination of this information through awareness and advertising campaigns [45, 46, 47] and reluctance to take oral supplements.

Patients with vitiligo showed the highest level of compliance with UV protection recommendations. In their examination of the perception of skin cancer risk and sun protection practices among individuals with vitiligo, Gonzalez et al. [48] reported that almost half of respondents believed they had an increased risk of skin cancer due to their vitiligo, and nearly a quarter believed phototherapy increased this risk.

On the other hand, a recent Delphi consensus [49] about personalized medical photoprotection reported that UV radiation effects are cumulative, increasing the long‐term risk of photoaging and photocarcinogenesis in patients receiving phototherapy, who are also exposed to natural sunlight. While broad UV protection is generally recommended, we propose that these measures be tailored to the specific treatment: stringent protocols are vital for PUVA, whereas standard sun‐safety practices may suffice for narrowband UVB [49, 50]. Notably, inconsistent UV protection should be addressed as a potential confounding variable that can obscure clinical outcomes.

No previous studies have specifically analysed and compared compliance with expert recommendations for UV protection in patients with cutaneous IMIDs. Assessing compliance is crucial to identify barriers to effective UV protection, tailor educational interventions, and ultimately improve overall patient outcomes.

### 4.3 UV Protection Preferences of Patients With Cutaneous IMIDs

Our study results demonstrate that UV protection preferences among patients with IMIDs are diverse, influenced by both the clinical characteristics of each disease and the product's comfort and practicality.

Most IMID patients preferred cream formulations for sun protection format. However, distinct preferences emerged across diseases, with alopecia areata (70%, *n* = 14) and HS (50%, *n* = 20) patients displaying a clear preference for sprays. This is likely due to its ease of application and reduced friction in sensitive or inflamed areas. These patients may also factor in facial application, where alcohol‐based spray formulations have been rated more favorably. This aspect was studied by Soky et al. [51], who conducted a study evaluating 139 participants' preferences for different facial sunscreen formulations using a randomized, blinded, half‐face application design. The main result was that participants significantly preferred alcohol‐based spray sunscreen over other formulations tested. This highlights the fact that the choice of format is influenced by the cosmetic appearance and sensory properties of the sunscreen [51, 52].

These differences show that format choice is not trivial, and that effective sunscreen strategy should consider individual preferences to enhance adherence.

Our findings highlighted specific barriers to sunscreen use among patients with atopic dermatitis. Factors such as thick texture, the presence of alcohol, perfumes, or preservatives, and sensations of stinging or itching after application significantly reduce their tolerance and regular use [1, 39]. Photosensitivity and the potential for developing contact allergy to certain filters, such as octocrylene [53], further complicate matters. These factors likely explain the lower degree of compliance with recommendations observed in this group compared to other IMID patients. Moreover, repeated sunscreen application to already damaged skin may be perceived as uncomfortable or even counterproductive by the patient due to potential absorption and systemic side effects, further diminishing adherence.

It is thus essential that sunscreen recommendations for IMID patients not only consider the required level of sun protection but also the product's format and tolerability, as well as the patient's subjective sensory perception.

### 4.4 Proposed Personalized UV Protection Recommendations for Patients With Cutaneous IMIDs

Based on expert recommendations and the preferences identified in this study, we propose the following measures to improve adherence to and effectiveness of UV protection in this patient group (Table 4):

- **Daily Use of Sunscreen:** Daily application of broad‐spectrum (UVB/UVA) sunscreen with high SPF, including UVB, UVA, and high‐energy visible light (HEVL) coverage, customized according to phototype (see below). Reapplication every 2 h during prolonged sun exposure and after sweating or swimming is essential [1, 3]. Special attention should be given to patients taking photosensitizing medications, such as tetracyclines, and also TNFα‐inhibitors and JAK‐inhibitors, considering the potential increased risk of cancer associated with these therapeutic groups in general [54, 55, 56].
- **Type of Filters:** Impaired skin barrier function is a feature of all cutaneous IMIDs. Therefore, avoiding filters like benzophenones, butyl‐methoxydibenzoylmethane, and octocrylene, which may cause allergic reactions (especially in patients with atopic dermatitis), is recommended [1, 51, 55, 56]. Safe organic filters and mineral‐based options (zinc oxide, titanium dioxide) are preferred [2, 57, 58].
- **Photosensitivity in Psoriasis and Atopic Dermatitis:** Very high UVB and UVA SPF, in addition to HEVL protection, is mandatory for these patients^1,8,10,^ for whom oral photoprotection may also be beneficial [18].
- **Cosmetic Acceptability and Format Preference:** Patient preferences should be acknowledged to improve adherence. For instance, ease of application and reduced friction make spray formulations particularly suitable for patients with HS and alopecia areata [51]. However, sprays should not contain titanium dioxide nanoparticles to avoid inhalation risks [2]. Creams may be more suitable for patients with atopic dermatitis and psoriasis, who typically moisturize their skin [38, 39, 59].
- **Physical Photoprotection:** Patients should be aware that physical protection, such as hats, caps, ultraviolet‐protective clothing, and sunglasses, is as important as sunscreen use, and that both should be used in parallel [1, 3]
- **Oral Photoprotection as an Adjuvant Strategy:** It is crucial to inform and educate patients about the existence of oral photoprotectors, especially for patients with vitiligo or psoriasis (where controlled sun exposure is recommended), those receiving phototherapy, and those who are highly photosensitive [17, 18]. In vitiligo and psoriasis patients, sun exposure should be limited and controlled to prevent sunburn, which could cause Koebnerization [1, 60].
- **Vitamin D:** Maintaining good serum vitamin D levels is particularly important for individuals with immune‐mediated diseases. Despite adapting protective measures to phototypes (see below), the impaired skin function and frequent vitamin D deficiency observed in these patients make vitamin D supplementation advisable to ensure healthy levels [35, 61, 62, 63].
- **Education and Personalized Follow‐up:** Dermatologists play a fundamental role in educating these patients, integrating this information into clinical visits as an integral part of dermatological treatment [21]. It is also important to address issues such as discomfort with certain products [39] or misconceptions about the effects of the sun. Regular follow‐up may be beneficial to assess adherence and reinforce healthy habits.
- **Adaptation to the Patient's Reality:** All recommendations should be adapted to personal circumstances. Occupational sun exposure is arguably the most significant factor, as it substantially increases the risk of photoaging and skin cancer [63, 64, 65]. Skin phototype is also a determinant for personalized UV protection: for darker skin, SPF 30+ sunscreen with a similar UVA‐PF and HEVL protection is recommended [66, 67]. For lighter phototypes, SPF > 50 with an SPF/UVA‐PF ratio of < 3 and, if possible, HEVL protection is recommended [1, 21, 68]

**TABLE 4:** Proposed personalized UV protection recommendations for patients with cutaneous IMIDs.

Characteristic	Recommendation	Level of evidence	Grade of recommendation
Broad‐spectrum protection	Balanced SPF with UVB, UVA and high energy visible light (HEVL) coverage, adapted to the patient's phototype	V	D
Application frequency	Every 2 h during long sun exposures and after sweating or swimming	V	D
Type of filters	Safe organic and mineral‐based (zinc oxide, titanium dioxide) filters	IIb	B
Avoidable ingredients	Benzophenones, butyl methoxydibenzoylmethane and octocrylene (allergic reactions)	IIIa	B
Format preference	Sprays for hidradenitis suppurativa and alopecia areata patients Cream for atopic dermatitis and psoriasis patients	IV	C
Physical photoprotection	Hats, caps, clothes and sunglasses are recommended as for the general population	V	D
Oral photoprotection	Especially for patients with vitiligo or psoriasis, those who receive phototherapy or have high photosensitivity	IV	C
Vitamin D	Vitamin D supplements are recommended if the serum levels are lower than 30 ng/mL	Ia	A
Special considerations	Sprays should not contain titanium dioxide nanoparticles to avoid inhalation risks	IIb	B
Photosensitizing medications	UV protection is specially recommend in patients under tetracyclines, TNFα‐inhibitors and JAK‐inhibitors	IIb	B

This proposal seeks not only to align with current clinical recommendations but also to address the practical needs and barriers faced by patients with cutaneous IMIDs, thereby promoting effective, personalized UV protection and greater adherence.

## 5 Limitations

The main limitation of this study is its relatively small sample size. Another consideration is the selection of the control group from companions of patients attending the dermatology consultation, which could potentially introduce selection bias.

## 6 Conclusions

This study demonstrates that patients with common cutaneous IMIDs generally do not adhere to expert recommendations regarding sun protection practices. Furthermore, these recommendations largely fail to consider patient preferences, which significantly compromises adherence. We propose that adapting UV protection recommendations to the real‐world needs of each patient's condition can substantially improve therapeutic adherence and help prevent disease flare‐ups and complications arising from inadequate sun exposure.

Considering both expert recommendations and patient preferences, we propose a specific list of customized recommendations tailored for the diverse real‐life scenarios of these individuals.

Together, these findings underscore the critical need for personalized education around UV protection, specifically tailored to the unique characteristics and needs of each condition, in order to promote self‐care among IMID patients and ultimately improve adherence, disease control and patient quality of life.

## Author Contributions

Z.A.‐B., T.G.‐C. and Y.G.‐C. contributed to the preparation and editing of the manuscript. All authors contributed to the article and have approved the submitted version.

All persons designated as authors participated in the work and take public responsibility for its contents.

## Funding

The authors have nothing to report.

## Disclosure

The authors declare that no Artificial Intelligence (AI) technologies were used in the preparation of this manuscript.

## Conflicts of Interest

The authors declare no conflicts of interest.

## Supporting information


**Data S1:** The supplementary material includes the various questionnaires completed by study participants: the photoprotection preference questionnaire and a questionnaire exploring adherence to disease‐specific UV protection recommendations. Both new questionnaires were specifically developed for this study.

## Data Availability

The data that support the findings of this study are available on request from the corresponding author. The data are not publicly available due to privacy or ethical restrictions.
