# Supplementary material for: UV Protection Habits and Preferences in Patients With Distinct Cutaneous Immune‐Mediated Inflammatory Diseases
Source: Photodermatol Photoimmunol Photomed. 2026 Mar 15;42(2):e70084. doi: 10.1111/phpp.70084 (PMC12989706; doi:10.1111/phpp.70084)
Supplement: Supplementary file 1 — Data S1: The supplementary material includes the various questionnaires completed by study participants: the photoprotection preference questionnaire and a questionnaire exploring adherence to disease‐specific UV protection recommendations. Both new questionnaires were specifically developed for this study. [file PHPP-42-e70084-s001.docx]

**SUPPLEMENTARY MATERIAL**

**PHOTOPROTECTION HABITS QUESTIONNAIRE**

1. **Do you apply sunscreen or sun protection daily?**

Yes / No

**2. In the past month, how many times have you applied sunscreen?**

- Every day
- More than 20 days
- More than 10 days but less than 20 days
- Between 5 and 10 days
- Fewer than 5 days
- None

3. **On the days you apply sunscreen, how often do you apply it?**

- Every 2 hours
- Twice a day
- Once a day

4. **When using sunscreen, what protection factor do you use?**

- I don’t know
- 2–15
- 16–29
- 30–50
- Over 50

**5. How many times have you experienced a sunburn in the past year?**

- None
- 1–2
- 3–5
- 6–10
- Over 10

**6. Do you think sun exposure is beneficial for your condition?**

Yes / No

**7. When exposed to the sun, do you feel your condition improves?**

- No, it even worsens
- No change, neither improves nor worsens
- Yes, it improves
- Yes, it improves significantly

8. **Do you regularly use a hat or cap?**

Yes / No

**9. What type of sunscreen format do you currently use?**

- Lotion
- Spray
- Cream
- Gel
- Oils
- Stick

**10. What type of sunscreen format would you prefer as most suitable for your condition?**

- Lotion
- Spray
- Cream
- Gel
- Oils
- Stick

**11. Do you experience any discomfort when applying sunscreen (eye stinging, irritation, acne, etc.)?**

Yes / No

**12. Have you heard of “oral sun protection” (sun protection pills)?**

Yes / No

**13. Do you use oral sun protection (sun protection pills)?**

Yes / No

**14. If not, would you like to use it? Do you think it could be beneficial for your condition?**

- Yes, I’d like to use it / No, I wouldn’t
- Yes, I believe it could be beneficial / No, I don’t think it could help

**QUESTIONNAIRE WITH PHOTOPROTECTION RECOMMENDATIONS FOR EACH PATHOLOGY**

- **PHOTOPROTECTION RECOMMENDATIONS FOR ATOPIC DERMATITIS**

After reading the photoprotection recommendations provided by experts for your condition, indicate which ones you follow:

- - Sunscreen should not be applied until skin lesions are treated and inflammation is resolved to avoid systemic absorption and photosensitization reactions.

Do you follow this recommendation? YES / NO

- - High temperatures and sweating can worsen redness or itching of atopic dermatitis lesions, so these situations should be avoided.

Do you follow this recommendation? YES / NO

- - Sunscreen should not be applied to moist, oozing, or eroded lesions resulting from severe scratching.

Do you follow this recommendation? YES / NO

- - Regular use of broad-spectrum sunscreens (SPF50+) is recommended to prevent photosensitivity and sunburn.

Do you follow this recommendation? YES / NO

- **PHOTOPROTECTION RECOMMENDATIONS FOR PSORIASIS**

After reading the photoprotection recommendations provided by experts for your condition, indicate which ones you follow:

- - Sun exposure can be beneficial for psoriasis patients (they often notice improvement during spring or summer), so regular sun exposure is recommended.

Do you follow this recommendation? YES / NO

- - However, sun exposure should be limited, and patients should avoid sunburns by applying sunscreen.

Do you follow this recommendation? YES / NO

- **PHOTOPROTECTION RECOMMENDATIONS FOR VITILIGO**

After reading the photoprotection recommendations provided by experts for your condition, indicate which ones you follow:

- - Patients with vitiligo are advised to regularly expose affected skin to the sun without sunscreen until vitiligo lesions start turning pink. Once the lesions turn pink, apply SPF50+ sunscreen to prevent sunburn.

Do you follow these recommendations? YES / NO

- **PHOTOPROTECTION RECOMMENDATIONS FOR HIDRADENITIS SUPPURATIVA**

After reading the photoprotection recommendations provided by experts for your condition, indicate which ones you follow:

- - SPF50+ sunscreen is recommended to avoid post-inflammatory hyperpigmentation ("brown spots") on areas with lesions.

Do you follow this recommendation? YES / NO

- - Answer only if you are being treated with Tetracycline-family drugs (Doxycycline, Minocycline...): One of the most common side effects of this antibiotic family is photosensitivity. During treatment, rigorous daily photoprotection with broad-spectrum sunscreen (SPF50+) is essential.

Do you follow this recommendation? YES / NO

- **PHOTOPROTECTION RECOMMENDATIONS FOR ALOPECIA AREATA**

After reading the photoprotection recommendations provided by experts for your condition, indicate which ones you follow:

- - Physical protection measures (hat, cap, etc.) and SPF50+ sunscreen are recommended for lesions exposed to direct sunlight and devoid of hair.

Do you follow this recommendation? YES / NO
